# IgE and non-IgE-mediated pathways in anaphylaxis

**DOI:** 10.1007/s00281-025-01056-7

**Published:** 2025-08-13

**Authors:** Margitta Worm, Kristijan Pazur, Payam Morakabati, Davender Redhu

**Affiliations:** Division of Allergy and Immunology, Department of Dermatology, Venerology and Allergy, Charité Universitätsmedizin Berlin, corporate member of Freie Universität Berlin and Humboldt-Universität zu Berlin, Berlin, Germany

## Abstract

Anaphylaxis is a severe, potentially life-threatening allergic reaction that can occur through both IgE- and non-IgE-mediated pathways. The classic IgE-mediated pathway involves allergen-specific IgE binding to FcεRI on mast cells and basophils, triggering degranulation and the release of inflammatory mediators. Non-IgE-mediated mechanisms, which are commonly associated with drug-induced reactions, at least in mice, involve the activation of the G-protein-coupled receptor (MRGPRX2), triggering mast cell degranulation in an IgE independent manner. Anaphylaxis can also be mediated through IgG immune complex interaction with Fc gamma receptors on various cell types, leading to mediator release. This review will describe current understanding of the pathomechanisms of anaphylaxis. Understanding these diverse pathways is crucial for accurate diagnosis, effective treatment, and prevention of anaphylaxis.

## Epidemiology and clinical features of anaphylaxis

Anaphylaxis is a potentially life-threatening acute rapid systemic allergic reaction. Estimates of anaphylaxis prevalence vary widely, with many studies suggesting an increasing trend, especially in developed nations. The lifetime prevalence of anaphylaxis has been reported to range from 1.6% to 5.1%, with an incidence rate of 42 per 100,000 person-years [1]. The estimated mortality rate for drug-induced anaphylaxis ranges from 0.05 to 0.51 per million people annually, while food-induced anaphylaxis has a rate of 0.03 to 0.32, and venom-induced anaphylaxis is estimated at 0.09 to 0.13 per million people per year [2].

A defining characteristic of anaphylaxis is its systemic nature, distinguishing it from localized allergic reactions. Upon triggering, there is a rapid and widespread release of potent mediators, such as histamine, tryptase, leukotrienes, and platelet-activating factor (PAF), into the circulation. This systemic dissemination allows these mediators to exert their effects on distant organs, leading to the characteristic multi-organ involvement seen in anaphylaxis. Unlike localized reactions where mediator effects are confined to the site of allergen exposure, the systemic release of mediators in anaphylaxis results in widespread vasodilation, increased vascular permeability, bronchoconstriction, and smooth muscle contraction in various tissues, ultimately manifesting as cutaneous symptoms, respiratory distress, cardiovascular collapse, and gastrointestinal disturbances that can lead to death [3].

Cutaneous symptoms, including urticaria, angioedema, erythema, and pruritus, are among the most common manifestations [4]. The signs and symptoms of anaphylaxis can be unpredictable and may vary in an age-dependent manner among individuals and reactions [5, 6].

### Etiology of anaphylaxis

**Fig. 1:**
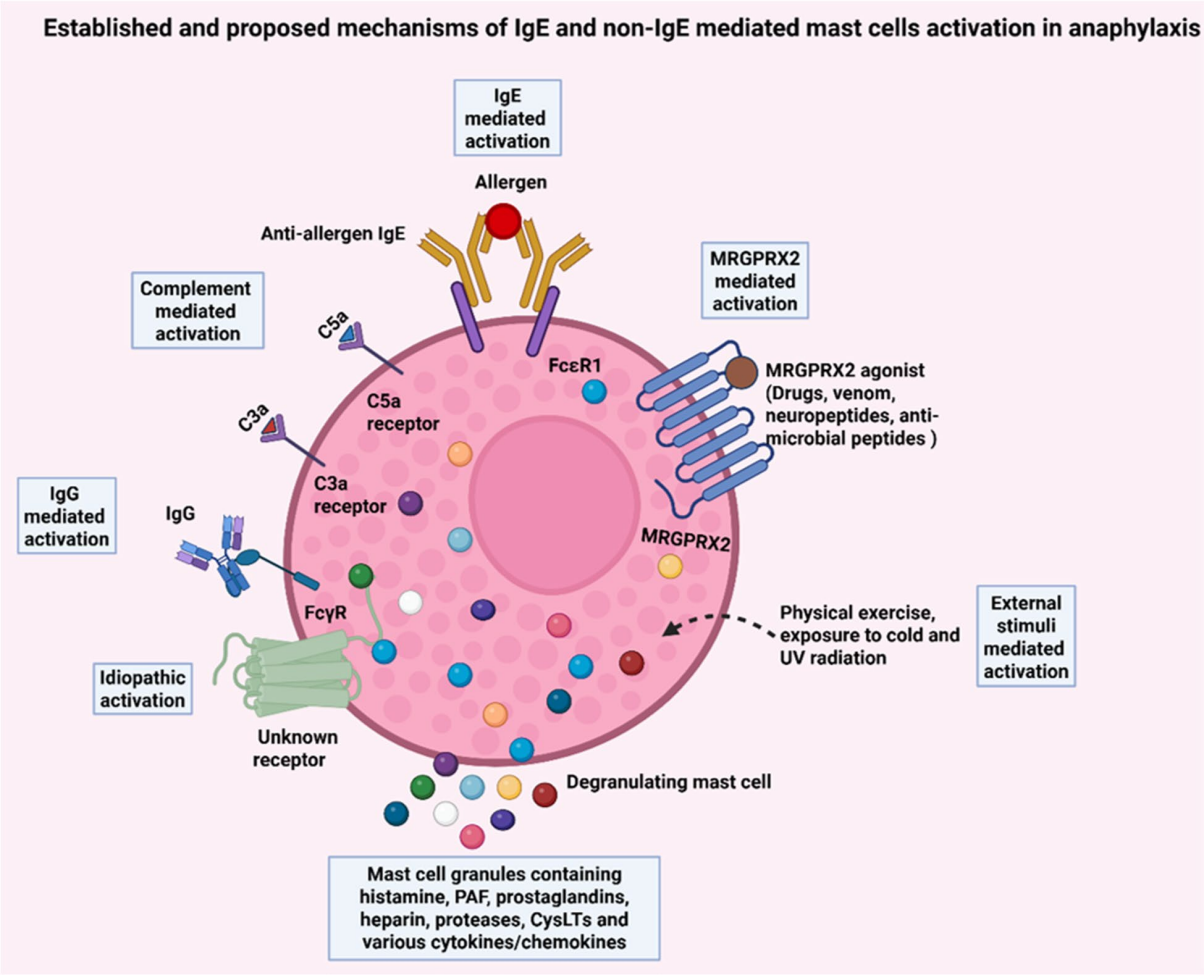
Graphical illustration of the various pathways of mast cells activation. Key mechanisms include: IgE-mediated activation: Interaction of allergens with anti-allergen IgE bound to FcεRI receptors, leading to degranulation. Complement-mediated activation: Activation via complement components (C3a and C5a) binding to their respective receptors on effector cells. IgG-mediated activation: IgG molecules binding to FcγR receptors, triggering cellular activation. MRGPRX2-mediated activation: Activation through MRGPRX2 agonists, such as drugs, venoms, neuropeptides, and antimicrobial peptides. External stimuli-mediated activation: Activation by physical triggers like exercise, cold exposure, and UV radiation that indirectly activate effector cells. Idiopathic activation: Activation through unknown pathway/allergen. Degranulation of effector cells results in the release of various granules containing inflammatory mediators (histamine, PAF, prostaglandins, heparin, proteases, CysLTs and various cytokines/chemokines), contributing to immune responses and allergic reactions. Created using Biorender

Anaphylaxis can be triggered by various stimuli. Most frequently it is mediated by allergen/antigen which results in the cross-linking of immunoglobulin E (IgE) leading to activation of mast cells and basophils and the subsequent release of inflammatory mediators. While drugs (antibiotics) and insect venom are the most frequent culprits in adults, food is the most common trigger of anaphylaxis in children and adolescents [1, 5]. While although less common, exercise, non-steroidal anti-inflammatory drugs (NSAIDs), opioids and radiocontrast agents can also induce anaphylaxis (Fig. 1). However, these reactions frequently involve non-IgE-mediated mechanisms. Moreover, in some instances, the underlying cause of anaphylaxis remains elusive, referred to as idiopathic anaphylaxis (Fig. 1) [5].

The severity of anaphylactic reactions is highly variable, often differing even when exposure to the same allergen and dose is repeated. Augmentation factors, or cofactors can exacerbate severity of reactions or even trigger responses to allergens that would otherwise be tolerated [7]. They can be categorized as intrinsic (host-dependent and often physiological, e.g. age, sex, concomitant atopic diseases and mastocytosis) and extrinsic factors (related to behavior or external influences, e.g. psychological burden, exercise, drugs including beta-blockers and ACE inhibitor) [8].

### Pathophysiology of anaphylaxis

#### IgE mediated anaphylaxis

The primary mechanism behind most cases of human anaphylaxis is mediated by classic type-I allergic reactions pathway involving IgE and mast cells/basophils. The initial pathogenic step in the development of IgE-dependent type I allergy is sensitization, a process mediated by the uptake of allergens by antigen-presenting cells (APCs). APCs capture and process allergens, presenting fragments on their surface via MHC class II molecules, undergo maturation, and migrate to regional lymph nodes, where they present these antigens to naïve T cells. This interaction is crucial for the differentiation of CD4 + T cells into Th2 cells which will promote the adaptive immune response [9].

The selective expansion of Th2 cells, and their secretion of cytokines (IL-4, IL-13) orchestrate the allergic inflammatory response. Th2 cytokines promote Th2 cell survival, mast cell differentiation and maturation, eosinophil maturation and survival, basophil recruitment and B cell activation and isotype switching [9]. T cells also provide necessary signals through CD40L, which interacts with CD40 on B cells. Together, these interactions drive B cells to undergo class switching, a process whereby B cells shift from producing other antibody classes (such as IgM or IgG) to producing IgE antibodies specific to the allergen. IgE production is critical for establishing the body's immunological"memory"of the allergen [9, 10]​​. Class switching is primarily facilitated by the activation-induced cytidine deaminase (AID) enzyme, which aids in DNA recombination necessary for class switching to IgE. Upon maturation, these IgE-producing B cells circulate throughout the body and can migrate to the bone marrow where they reside as long-lived plasma cells. Circulating IgE molecules bind to high-affinity FcεRI receptors on the surfaces of mast cells and basophils, priming these cells for future exposure to the allergen [10, 11]​. During the effector phase, re-exposure to the same allergen cross-links the IgE molecules bound to FcεRI receptors on mast cells and basophils (Fig. 1). This cross-linking initiates a rapid intracellular signaling cascade within these cells. Lyn-dependent phosphorylation of ITAMs on FcεRI leads to Syk activation and autophosphorylation. Syk subsequently phosphorylates LAT and NTAL, initiating downstream signaling cascades that involve PLCγ, PI3K, calcium influx, and PKC activation. Sufficiently robust signaling activates the mast cell or basophils that culminate in degranulation and the release of preformed mediators, including histamine, tryptase, chymase, carboxypeptidase A3, TNF-α and heparin. Additionally, there is fast subsequent release of newly synthesized mediators like LTC4, LTD4, LTE4, PAF and PGD2. The release of mediators, results in local vasodilatation, edema formation, neurogenic stimulation, and mucus secretion. During the late phase, mast cells synthesize and secrete numerous cytokines and chemokines including: TNF-α,IL-1, IL-13, IL-4, IL-3, GM-CSF, IL-6, IL-10, IL-5, CXCL8 and CCL3 that facilitate the activation of local cells and the recruitment of inflammatory cells, including granulocytic leukocytes (neutrophils, basophils, eosinophils) and agranular leukocytes (monocytes, lymphocytes), to the site of inflammation [9, 10, 12–14].

#### IgG mediated anaphylaxis

Despite experiencing anaphylactic reactions, ~ 30–50% of patients with drug induced anaphylaxis and ~ 20–30% of patients with food induced anaphylaxis exhibit no detectable levels of allergen-specific IgE [15, 16]. This suggests that a subgroup of individuals may not exhibit IgE-dependent immune activation, implying the involvement of alternative cellular and molecular mechanisms. Given the similarities between mouse and human immune systems, murine models have been instrumental in elucidating these alternative pathways [17]. Among them, the IgG pathway has emerged as a prominent mechanism in anaphylactic reactions.

IgG-class antibodies bind to Fc gamma receptors (FcγRs) on various cell types, initiating different signaling pathways. FcγRI, FcγRIIA, FcγRIIC, FcγRIIIA, and FcγRIIIB all provide activating signals, while FcγRIIB provides inhibitory signaling. FcγRI is unique in its high-affinity binding to monomeric IgG (particularly IgG1, IgG3 and IgG4) and its expression on both mast cells and neutrophils. Binding of specific IgG1 to FcγRI on mast cells can lead to cell activation in the presence of allergen. Both IgG1 and IgG3 can activate FcγRI on monocytes and macrophages [18].

Unlike the IgE dependent pathway, the IgG mechanism appears to require higher amounts of specific IgG and antigens, likely due to the lower affinity of FcγR for IgG compared to FcεRI for IgE. It is now well-established that IgG antibodies can induce anaphylaxis in mice by binding to their respective receptors (FcγR), which are expressed on mast cells, basophils, neutrophils, monocytes, and macrophages. These interactions can lead to cellular activation and the release of abundant mediators. One common mediator released in response to both IgE and IgG molecules is PAF, which is produced by various myeloid progenitor cell lineage subsets [17, 19, 20]. Recent publications suggest the involvement of IgG in human anaphylactic reactions, especially in neuromuscular blocking agents (NMBAs) drug-induced cases [15, 21]

These authors show elevated anti-NMBA IgG titers and neutrophil activation, correlating with increased PAF levels compared to non-anaphylactic controls. Their proposed mechanism involves IgG complex engagement of neutrophil FcγRIIA and FcγRIII, triggering PAF release. Notably, 31% of this cohort lacked IgE-associated biomarkers, underscoring that IgG-mediated anaphylaxis, can manifest independently of IgE hypersensitivity. Clinical observations, such as anaphylaxis in patients with therapeutic-specific IgG antibodies but undetectable IgE, and the association of FcγRIIA polymorphisms with anaphylaxis in specific populations, further support this hypothesis [22]. However, alternative explanations, such as the presence of low-level IgE or the role of other receptors or inflammatory mediators, cannot be entirely excluded. Further research is needed to definitively establish the role of IgG in human anaphylaxis [17, 19].

#### Immunoglobulin independent mast cell activation

Non-IgE-mediated mast cell activation is a recognized mechanism contributing to anaphylaxis-like symptoms independent of IgE pathways. Among the G-protein-coupled receptors implicated in this process, the Mas-related G-protein-coupled receptor X2 (MRGPRX2) has gained attention for its role in drug-induced pseudo-allergic reactions. However, its relevance to human anaphylaxis remains largely speculative, as direct evidence of MRGPRX2 activation in clinical anaphylaxis is currently lacking [23].

MRGPRX2 is selectively expressed on human mast cells and responds to a variety of cationic ligands, including some antimicrobial peptides, neuropeptides, and selected FDA-approved drugs like neuromuscular blocking agents and fluoroquinolones [17, 18, 24, 25]. Activation of this receptor can induce mast cell degranulation and mimic allergic symptoms such as flushing, urticaria, and hypotension, without IgE involvement [26, 27]. However, for many of these drugs, the concentrations achieved in human plasma during therapeutic use are insufficient to activate MRGPRX2, raising questions about its physiological relevance in vivo.

Much of the understanding of MRGPRX2's function derives from studies in murine models that utilize its rodent ortholog, Mrgprb2. Importantly, MRGPRX2 and Mrgprb2 differ significantly in their ligand specificity and sensitivity, and data from mouse models do not directly translate to human physiology [28, 29]. For instance, several compounds that activate Mrgprb2 do not bind or activate MRGPRX2 at clinically relevant doses in humans [30]. Thus, while murine studies have shown that Mrgprb2 is involved in non-IgE-mediated mast cell responses to drugs like atracurium and ciprofloxacin, the human *in-vivo* relevance of these findings remains unproven [28–33]. Ongoing and upcoming clinical trials (NCT06077773, NCT06050928) evaluating MRGPRX2 antagonists in chronic spontaneous and inducible urticaria will clarify the receptor's role in mast cell–mediated disease.

In addition to MRGPRX2 other pathways that bypass IgE have been investigated. For instance, complement activation, particularly the release of anaphylatoxins such as C3a and C5a, can directly activate mast cells and contribute to anaphylactic reactions (Fig. 1) [34, 35]. These complement fragments bind to their respective receptors on mast cells and trigger degranulation, leading to the release of histamine and other mediators. Complement-mediated anaphylaxis can occur in response to certain drugs, particularly those administered intravenously, such as liposomal formulations and monoclonal antibodies [36, 37]. Future research should continue to explore the interplay between these alternative mechanisms and mast cell activation in human anaphylaxis.

#### Biomarkers of anaphylaxis: classical and novel insights

Anaphylaxis, a rapid and life-threatening allergic reaction, presents a diagnostic challenge due to its variable clinical manifestations and the need for rapid therapeutic intervention. Accurate and timely diagnosis, coupled with monitoring for potential relapse, has spurred research into biomarkers that could aid in identifying, predicting, and assessing the severity of anaphylactic events. Biomarkers in anaphylaxis serve various roles, from confirming the occurrence of the reaction to providing insight into the underlying pathophysiology. Effector cells, receptors/ligands and mediators are summarized in Table 1.

**Table 1:** Highlighting the effector cells, receptors/ligands, and mediators/biomarkers involved in anaphylactic reaction

Effector cells	Receptors/ligands	Mediators/biomarkers
Mast cell	Constitutive expression: FcεRI [38–40], MRGPRX2 [38, 39, 41], C3aR [39, 42–44], C5aR [38, 39, 42], PAF-R[38, 39, 45], TLR 1–7,9 [39, 46], IL-33R [39, 47] Inducible under inflammatory condition: FcϵRII (CD23) [39, 48], FcγRI [38, 49], FcγRIIa [39, 49], TSLPR [39, 50]	Tryptase [12, 38, 39, 51, 52], Histamine [12, 38, 39, 51], Chymase [39, 51], Heparin [39], PAF [53–55], PAF-AH [39], LTC4 [54], LTD4 [54], LTE4 [39, 54], PGF2 [54], PGD2 [51], PGE2 [51], TNF-α [51, 56], TGF-β [51], IL-2 [39], IL-4 [39, 51], IL-5 [51], IL-6 [39, 51, 56], IL-8 [56], IL-10 [39], IL-13 [51, 56], IL-33 [51], CPA3 [51], GM-CSF [51], CCL-2 [51], SCF [39], TXA2 [39], TXB2 [39]
Basophil	Constitutive expression: FcεRI [38, 39, 57], FcγRIIa [12, 39, 58], C3aR [39, 43, 44], C5aR [39, 59], TLR2,4,9,10 [39, 60]	Tryptase [39, 51], Histamine [39, 51, 54], Chymase [39], Heparin [39], PAF [61], LTE4 [39], IL-4 [39], IL-6 [39]
Eosinophil	Constitutive expression: FcϵRII [39], FcγRIIa [39, 62], C3aR [39, 62], C5aR [39, 62], TNFR1 [39, 63]. IL-33R [39, 64] Inducible under inflammatory condition: FcϵRI [39, 65], FcγRIII [54, 66], TLR1-5,7,9 [39, 60, 67]	Histamine [39], Nitric oxide, [54], PAF [51, 61], MBP [26, 56], PGE2 [51], TGF-β [51], LTE4 [39], IL-4 [39, 51], IL-5 [51], IL-10 [39], IL-13 [51], TXA2 [39]
Neutrophil	Constitutive expression: FcγRIIA, FcγRIIIB [68], C5aR, C3aR [43], PAF- [39], G-CSFR [39], GM-CSFR [39], TNFR[39], IL-1R [39] Inducible under inflammatory conditions: FcγRI, FcγRIIB [68], FcϵRI [39]	PAF [51, 54], Elastase [51], MPO [12, 39, 51], LTB4 [39, 51], LTC4 [51], LTD4 [51], LTE4 [51], TNF-α [51], IL-6 [39, 51], CCL-2 [51]
Platelet	Constitutive expression: FcγRIIa [69], TLR1,2,4,6 [70], C1qR [70], C3aR [70]	PAF [51, 61], PAF-AH [39], PGD2 [51], PGE2 [51], PGF2 [51], TXA2 [39]
Macrophage	Constitutive expression: FcγRIII [71], FcγRIIA [71], C3aR [72], C5aR [39], TNFR [73], TLR1,2,4,5,7,9 [39, 74] Inducible under inflammatory conditions: FcγRI [71], FcϵRI [75]	PAF [51, 53, 61, 76], PAF-AH [39], MPO [51], LTE4 [39], PGD2 [51], PGE2 [51], TNF-α [51], IL-6 [39, 51], CCL-2 [51]

Abbreviatios: *FcϵRI* high affinity IgE receptor, *FcϵRII (CD23)* low affinity IgE receptor, *FcγRI* Fc-gamma receptor 1, *FcγRIIa(CD32)* Fc-gamma receptor 2a, *FcγRIII(CD16)* Fc-gamma receptor 3, *MRGPRX2*, Mas-related G protein-coupled receptor X2, *PAF-R* plasmin activator factor receptor, *TLR* Toll-like receptor, *TSLPR* thymic stromal lymphopoietin receptor, *TNFR* tumor necrosis factor receptor, *GCSFR* granulocyte colony stimulating factor receptor, *GM-CSFR* granulocyte–macrophage colony-stimulating factor receptor, *LTC, D, E4* leukotrienes C, D, E4, *PGD2* prostaglandin D2, *PGE2* prostaglandin E2, *PGF2* prostaglandin F2, *TGF-β* transforming growth factor beta, *TNF-α*Tumor necrosis factor-alpha, *CPA3* Carboxypeptidase A3, *GM-CSF* Granulocyte–macrophage colony-stimulating factor, *CCL-2* C–C motif chemokine ligand 2, *SCF* Stem cell factor, *TXA2* thromboxane A2, *TXB2* thromboxane B2, *PAF* Platelet-activating factor, *PAF-AH* PAF acetylhydrolase, *MPO* myeloperoxidase, *MBP* Major basic protein.

Tryptase, a serine protease predominantly released from mast cells, although basophils and myeloid precursors contain smaller amounts. Currently, it is the main biomarker of anaphylaxis used in clinical practice. Its concentration peaks within 1–2 h of anaphylaxis onset and declines over the next 6 h. Due to high interindividual variability distinguishing the relevant increase of tryptase upon allergic reaction require serum tryptase levels measurement at baseline and during a reaction. The consensus equation (peak tryptase > 1.2 × baseline tryptase + 2 ng/L) has been proposed to determine acute tryptase elevations [77, 78]. An increased acute tryptase levels have been shown to correlate with severity [79, 80]. However, systemic tryptase levels may not increase in all cases of anaphylaxis, e.g. in food-induced reactions. Elevated levels of tryptase at the baseline are associated with mast cell activation disorders like hereditary α tryptasemia and mastocytosis [81].

Tryptase exists in two biologically relevant forms: alpha-protryptase and beta-tryptase. Alpha-protryptase is an inactive proenzyme secreted constitutively into the serum. In contrast, beta-tryptase is the enzymatically active form, a mature tetramer stabilized by proteoglycans. It is predominantly stored in mast cell secretory granules and released upon mast cell activation. Current commercial methods measure both alpha and beta tryptase; however, for optimal diagnosis of anaphylaxis, measuring beta-tryptase alone is preferable, as it is the form released by mast cells [39, 82].

Histamine is prestored in granules of MCs and basophils. It acts through four distinct receptors (H1, H2, H3, and H4), which are widely distributed across various cell types. Upon activation, these receptors initiate a cascade of intracellular signaling pathways, ultimately leading to vasodilation, increased capillary permeability, bronchoconstriction, itching, and other symptoms [83]. Histamine, despite its important role in anaphylaxis, is limited as diagnostic biomarker in routine clinical practice due to its rapid enzymatic degradation into two metabolites: N-methylhistamine and N-methylimidazole octane. These substances can be found in urine for up to 24 h after the reaction begins. While urine testing is less invasive than blood tests sensitivity and specificity is significantly lower [39, 84].

Lipoxygenase products, such as leukotriene E4 (LTE4), are synthesized de novoby activated mast cells, neutrophils, eosinophils, and macrophages. LTE4, the most stable metabolite of lipoxygenase products, binds to CysLTR1, CysLTR2, and CysLTR3 receptors, leading to airway constriction, smooth muscle contraction, and increased vascular permeability. While LTE4 can also be measured in urine, its precursors LTC4 and LTD4 are rapidly metabolized and cannot be detected.

Cyclooxygenase products, including prostaglandins and thromboxanes, are also generated during anaphylaxis. PGD2, the primary cyclooxygenase product formed by mast cells, is involved in increasing vascular permeability and inducing bronchoconstriction. Although PGD2 is rapidly degraded, its more stable metabolites, like 9α,11β-PGF2 may serve as potential biomarkers for anaphylaxis [12, 51, 85, 86].

Platelet-activating factor (1-O-alkyl-2-acetyl-sn-glycero-3-phosphocholine), a potent phospholipid mediator, very similar to histamine in its effect, but more potent as it acts at much lower concentrations. It is produced by a variety of cells, including neutrophils, mast cells, endothelial cells, eosinophils, macrophages, monocytes and platelets. During anaphylaxis, PAF binds to its receptor, a member of the GPCR superfamily, that is widely expressed in various cells and tissues involved in inflammation and immune responses as well as cardiovascular, respiratory, and nervous system regulation [18]). This leads to bronchoconstriction, vasodilation and edema. Additionally, PAF is promoting in platelet aggregation and eosinophil activation [39]. Elevated PAF levels have been shown to correlate with the severity of anaphylaxis [53]. Moreover, an inverse correlation between PAF and PAF-acetylhydrolase activity has been demonstrated. PAF is commonly observed in both IgE- and IgG-mediated anaphylaxis due to its release by all myeloid cell subsets. One of the challenges to measure PAF in clinical practice is its short half-life. [12, 39, 51].

Beyond the above discussed classical biomarkers, molecules such as microRNAs (miRNAs) are a novel class of potential biomarkers. MicroRNAs are small, non-coding RNAs that regulate gene expression and have been implicated in immune system modulation [87, 88]. Dysregulated miRNA profiles, including miR-451a, miR-375-3p, have been recently identified in anaphylaxis. These offer promising new avenues for diagnostic and prognostic applications in allergology including anaphylaxis [86, 89].

While these classical and novel biomarkers offer valuable tools to improve the diagnostic accuracy and the mechanism of anaphylaxis, ongoing research will refine their clinical applications and enhance their reliability across diverse patient populations.

## Pathogenesis-based treatments

### The critical first-line therapy

#### Epinephrine

Adrenaline (epinephrine) is the first linedrug for treating acute anaphylaxis. Its rapid, pharmacologic action with cardiovascular and respiratory stabilizing action [90–92]. It is administered intramuscularly (IM), with a weight dependent-standard dosing between 0.15–0.5 mg every 5–10 min, as needed, based on the clinical response. It reaches the peak plasma concentration after administration within minutes, offering a fast intervention against the life-threatening symptoms of anaphylaxis [93, 94]. The alpha-1 adrenergic receptor agonism of epinephrine causes vasoconstriction, reversing peripheral vasodilation and maintaining blood pressure [95]. The beta-1 adrenergic receptor increase heart rate and contractility, countering anaphylactic bradycardia and ensuring adequate organ perfusion. Beta-2 adrenergic receptors stimulation by adrenaline in airways leads to bronchodilation and inhibits mast cell mediator release, relieving respiratory symptoms [95–97]. The plasma half-life of epinephrine is short (~ 1–3 min), though clinical effects last longer [98, 99]. This necessitates repeated dosing or continuous infusion in severe or persistent cases, especially when co-factors like beta-blockers are involved.

By acting on mast cells, epinephrine reduces the release of histamine and other mediators, addressing the root cause of the allergic cascade and helping to prevent further deterioration of the patient’s condition [100].

## Second-line agents for symptom relief

### Antihistamines

Antihistamines, although its limited role in the treatment of anaphylaxis but are often employed as second-line agents to manage cutaneous symptoms such as hives and swelling, which are caused by the release of histamine [96, 101, 102]. H1 antihistamines block H1 receptors, reducing itching, vasodilation, and bronchoconstriction [83, 102–104]. Diphenhydramine and cetirizine are common agents for skin, nose, and respiratory symptoms, but are ineffective for severe anaphylaxis [1, 82, 96]. H2 blockers (ranitidine, famotidine) inhibit H2 receptors, primarily in the stomach and vascular endothelium [103, 105]. Combining H1 and H2 blockers may enhance symptom relief, especially for gastric and cardiovascular effects [105, 106].

### Corticosteroids

Although corticosteroids have no immediate effect in the acute management of anaphylaxis but play a key role in long-term control of inflammation [96, 107–109]. Corticosteroids, like prednisone, methylprednisolone, and dexamethasone, inhibit the activation of NF-κB and other transcription factors, leading to a reduction in pro-inflammatory cytokine production (e.g., IL-1, TNF-alpha, IL-6) [110]. These drugs stabilize cell membranes, including those of mast cells, and reduce further mediator release during the delayed phase of anaphylaxis [111–113].

### Bronchodilators

Bronchodilators, such as albuterol (salbutamol), are used to treat bronchospasm that occurs during anaphylaxis, particularly when epinephrine alone does not completely resolve the respiratory distress [114]. Albuterol, a beta-2 adrenergic agonist, promotes bronchodilation by relaxing the smooth muscles in the airways. This improves oxygenation and reduces wheezing and respiratory difficulty [115]. While bronchodilators are valuable in reversing bronchospasm, they do not address the systemic immune-mediated effects of anaphylaxis. Therefore, they are used as an adjunct to epinephrine rather than a primary treatment [114].

### Glucagon

Patients on beta-blockers may be resistant to the effects of epinephrine due to the drug's blockade of beta-adrenergic receptors. In such cases, glucagon can be used as an alternative to support cardiovascular function [96, 116]. Glucagon bypasses beta-receptors by activating glucagon receptors, leading to increased cyclic AMP in cardiac cells. This enhances heart rate and myocardial contractility, thus restoring blood pressure and improving perfusion even when beta-receptors are blocked [117, 118]. Glucagon is reserved for patients who are on beta-blockers and have severe refractory anaphylaxis that does not respond to epinephrine [96].

### Vasopressors

In cases where patients remain hypotensive despite administration of epinephrine and IV fluids, vasopressors such as norepinephrine and vasopressin are used to restore vascular tone and improve blood pressure [119]. Norepinephrine works by stimulating alpha-1 adrenergic receptors, leading to potent vasoconstriction. It is administered via a continuous IV infusion in severe cases of refractory anaphylactic shock [96, 120]. Vasopressin, an antidiuretic hormone, causes vasoconstriction via V1 receptors on smooth muscle cells in the vasculature. It can be used alongside norepinephrine to further stabilize blood pressure in refractory cases [121, 122].

### Allergen avoidance

Allergen avoidance is the most straightforward preventive strategy in anaphylaxis, though it can be challenging due to the ubiquity of certain allergens, particularly in cases involving food or environmental triggers [123]. Identifying specific allergens and limiting exposure in everyday settings is the first line of defense against anaphylaxis. The ability to accurately identify an allergen is critical for preventing anaphylaxis. This may involve skin testing, serum-specific IgE tests, or oral food challenges to pinpoint the causative allergen. Once the allergen is known, strict avoidance is the goal.

### Immunotherapy

Allergen specific immunotherapy (AIT) is a treatment with increasing exposure to an allergen in controlled amounts to modify the patient’s immune response [124, 125]. The reduction or elimination of symptoms is the goal of AIT. The routes of AIT depend on the age, severity and availability of the allergen extract.

Most widely it is given subcutaneous (subcutaneous immunotherapy, or SCIT) or sublingual (sublingual immunotherapy, or SLIT). The immune response is shifted within the course of AIT from an IgE-mediated allergic reaction toward a more regulated immune response, potentially involving increased production of regulatory T cells or increased production of blocking antibodies like IgG4 [126].

Oral immunotherapy (OIT) is an emerging approach for IgE-mediated food allergies, aiming to increase the threshold of reactivity through controlled allergen exposure. Studies show OIT can achieve desensitization in 68–84% of children, particularly for peanut, milk, and egg allergies. While it may reduce anaphylaxis risk, adverse events—including systemic reactions and eosinophilic esophagitis—are common [127]. OIT requires specialist supervision and is recommended only for selected patients, with careful shared decision-making due to its risks and burden [128].

### Use of biologics

Omalizumab, a monoclonal antibody that targets IgE, is one of the most studied biologics as a novel preventive strategy for patients at high risk of anaphylaxis [129, 130]. It can be used to prevent allergic reactions by binding to IgE, thereby preventing the interaction of allergens with mast cells and basophils, which are responsible for the release of histamine and other mediators causing anaphylaxis. By reducing free IgE in the circulation, omalizumab reduces the sensitivity of cells to allergens, thereby decreasing the onset of anaphylactic reactions. Beyond neutralizing free circulating IgE, omalizumab can also accelerate the dissociation of IgE already bound to FcεRI on mast cells and basophils, leading to receptor internalization and downregulation. This effect contributes to a more rapid reduction in effector cell sensitivity than previously valued [130–133]. Multiple studies have demonstrated the effectiveness of omalizumab in reducing the risk of anaphylaxis in patients with food allergies and in those undergoing AIT [134, 135]. Additionally, real-world evidence supports its use in managing refractory anaphylaxis, where patients do not respond to standard treatments [129, 136, 137]. While omalizumab has proven to be effective in preventing allergic reactions, it does have limitations. Common side effects include local injection site reactions, while rare but serious side effects include anaphylaxis to the drug itself [138].

Disruptive IgE inhibitors, a novel class of engineered anti-IgE antibodies. Unlike omalizumab, which primarily sequesters free IgE, these molecules actively dislodge pre-bound IgE from FcεRI, leading to faster desensitization of allergic effector cells. Preclinical studies in murine models have shown that such agents can even abort ongoing anaphylactic reactions by rapidly disrupting IgE-FcεRI interactions [139–141]. These disruptive antibodies represent a potential promising therapy in both prophylactic and therapeutic applications for severe allergic diseases, although they remain in early-stage development.

### Investigational therapies

Bruton’s Tyrosine Kinase inhibitors (BTKis) have emerged as a promising therapeutic approach for IgE-mediated anaphylaxis. BTK is broadly expressed across a wide array of immune cell types, including mast cells, basophils, B lymphocytes, natural killer cells, macrophages, neutrophils, dendritic cells, monocytes, and osteoclasts [142–144]. BTKis primarily interfere with signal transduction downstream of the FcεRI receptor, thereby inhibiting mast cell and basophil degranulation in response to allergen-IgE complexes. However, these inhibitors do not alter the IgE sensitization state or antigen-specific memory, and thus do not target upstream immune priming or IgE production. Recent phase II clinical trial data have shown that acalabrutinib, a BTKi, effectively blocks mast cell and basophil activation, preventing the release of inflammatory mediators and potentially reducing the severity of anaphylaxis [145]. Acalabrutinib inhibits Bruton’s Tyrosine Kinase a key enzyme that drive IgE mediated mast cell and basophil degranulation [145, 146]. Besides its use in food allergies, BTKis have shown promise in managing drug-induced anaphylaxis. Recently, ibrutinib (BTKi) was successfully used to prevent anaphylactic reactions during drug desensitization in a patient undergoing chemotherapy [147]. This demonstrates the potential use of BTKis in preventing drug-related allergic reactions, particularly in settings where patients are at high risk. These findings highlight the rapid onset of action of BTKis, which make them useful in high-risk allergic patients. Another BTKi, remibrutinib has demonstrated sustained efficacy in chronic spontaneous urticaria [148, 149]. Its rapid onset of action and long-term benefits with fewer off-target effects compared to glucocorticoids, make it a promising candidate for widespread use in allergy management. Early-generation BTKis, such as ibrutinib, were associated with significant cardiovascular (e.g., atrial fibrillation, hypertension) and bleeding complications, due in part to off-target inhibition of kinases like TEC and ITK. Second-generation BTKis like acalabrutinib and zanubrutinib exhibit improved selectivity for BTK, which translates to a reduced incidence of atrial fibrillation and hypertension, as confirmed in head-to-head trials [150].

Epinephrine remains the gold standard for treating acute anaphylaxis, but it does not offer protection. Corticosteroids and antihistamines are limited in their ability to fully suppress IgE-mediated reactions, often leaving patients vulnerable to recurrent allergic episodes. In contrast, BTKis provide a preventive mechanism, acting upstream in the allergic cascade by inhibiting mast cell and basophil activation. This makes this new class of compound a valuable adjunct to existing therapies, particularly for patients who experience severe, recurrent anaphylaxis or require rapid desensitization protocols.

## Future direction and conclusions

As our understanding of anaphylaxis mechanisms advances, emerging therapeutic approaches such as BTKis show great promise. The role of BTK in IgE-mediated pathways opens possibilities for a more targeted anaphylaxis treatment. Early studies have shown that these inhibitors prevent mast cell and basophil activation, which are pivotal in the allergic cascade. If clinical trials in humans validate their efficacy, BTKis have the potential to revolutionize the treatment landscape, especially for patients with severe or recurrent allergic reactions. [145, 146, 149].

Furthermore, it is crucial to highlight that the majority of research on MRGPRX2 antagonists is currently limited to murine models. The efficacy and safety of MRGPRX2 antagonism in human is still unproven, and more extensive clinical trials are required to evaluate their potential as therapeutic agents in human anaphylaxis management. Until these trials are completed, MRGPRX2 antagonists remain a promising yet experimental option in the field of allergy and immunology.

In parallel with these advances, biologics like omalizumab are offering reliable protection for individuals at risk of IgE-mediated anaphylaxis. AIT, when combined with biologics, has also a high potential in building long-term tolerance to allergens. Adjunct therapies could improve the safety and efficacy of desensitization protocols, providing more comprehensive protection for patients at high risk of life-threatening allergic reactions.

Future research will also need to focus on personalized medicine approaches, as genetic factors such as mutations in MRGPRX2, FcγRs and differences in immune response pathways may dictate a patient’s susceptibility to certain allergens or treatments. Biomarkers like tryptase and MRGPRX2 receptor subtypes might be instrumental in tailoring diagnostics and individualized treatments, paving the way for more accurate and effective management of anaphylaxis.

In conclusion, while current symptomatic treatment strategies—such as epinephrine, antihistamines, and corticosteroids—remain critical in acute management of anaphylaxis treatment in the future may offer preventive strategies targeting the molecular underpinnings of allergic reactions. BTKis, MRGPRX2 antagonists, and biologics are the forefront of this advancement, offering novel approaches for better patient outcomes. As clinical trials advance, the adoption of these therapies can redefine anaphylaxis management, reducing both the incidence and severity of reactions.

## Data Availability

Data sharing not applicable to this article as no datasets were generated or analyzed during the current study.
